# Cellular Heterogeneity and Cooperativity in Glioma Persister Cells Under Temozolomide Treatment

**DOI:** 10.3389/fcell.2022.835273

**Published:** 2022-05-25

**Authors:** Marion Rabé, Lucie Fonteneau, Lisa Oliver, Alvaro Morales-Molina, Camille Jubelin, Javier Garcia-Castro, Dominique Heymann, Catherine Gratas, François M. Vallette

**Affiliations:** ^1^ Université de Nantes, INSERM U1232, CRCINA, Nantes, France; ^2^ CHU Nantes, Nantes, France; ^3^ Cellular Biotechnology Unit, Instituto de Salud Carlos III, Madrid, Spain; ^4^ Institut de Cancérologie de l'Ouest-St Herblain, Saint-Herblain, France

**Keywords:** glioma, persisters, clones, resistance, barcoding

## Abstract

We have observed a drug-tolerant/persister state in a human glioblastoma (GBM) cell line after exposure to temozolomide, the standard-of-care chemotherapeutic agent for GBM. We used a multicolor lentiviral genetic barcode labeling to follow cell population evolution during temozolomide treatment. We observed no change in the distribution of the different colored populations of cells in persister or resistant cells suggesting that pre-existing minor subpopulations, which would be expected to be restricted to a single color, were not amplified/selected during the response to the drug. We have previously identified four genes (*CHI3L1*, *FAT2*, *KLK5*, and *HB-EGF*) that were over-expressed during the persister stage. Single-cell analysis of these four genes indicated that they were expressed in different individual cells ruling out the existence of a single persister-specific clone but suggesting rather a global answer. Even so, the transitory silencing of *CHI3L1*, *FAT2*, or *KLK5* influenced the expression of the other three genes and the survival of U251 cells in absence of temozolomide. Since proteins encoded by the four genes are all localized in the extracellular matrix or interact within the extracellular compartment, we propose that cellular interactions and communications are important during the persister stage before the acquisition of chemo-resistance. Thus, persisters might be a new therapeutically relevant target in GBM.

## Introduction

In bacterial biofilms, antibiotic exposures have been shown to induce a tolerance/persister state under which bacteria can survive the treatment prior to developing specific resistance mechanisms (Levin-Reisman et al., 2017). Recently, a similar situation has been described in cancer exposed to tyrosine kinase inhibitors (TKI) in which resistance appears to rely on a non-specific drug-tolerant stage that precedes the emergence of cells expressing a mutant tyrosine kinase insensitive to TKI (Hata et al., 2016; Ramirez et al., 2016). In cancer, the mechanisms by which these persister cells acquire a tolerance to drugs are not well characterized and appear to be in multiples with more or less similarities with dormancy or cancer stem cell properties (Vallette et al., 2019; Oliver et al., 2020). However, several processes have been ascribed to persisters including epigenetic and metabolic reprogramming as well as cell-autonomous and non-autonomous processes between cancerous and/or environmental tumor cells (Swayden et al., 2020).

We have studied the process by which the U251 cell line, derived from human glioblastoma (GBM), acquired resistance to temozolomide, the current chemotherapy for GBM, through the expression of the DNA repair protein, O6-methylguanine-DNA methyl-transferase (MGMT) (Rabé et al., 2020). We have identified a transient state that followed temozolomide-induced cell death in U251 cells in which surviving slow-cycling cells were tolerant to high concentrations of temozolomide but do not express MGMT (Rabé et al., 2020). This state is highly reminiscent of the persister state described in GBM (Liau et al., 2017). In our previous study, we have determined a four-gene signature characteristic of the persister state. In this follow-up study, we have analyzed the interaction between the four genes both at the cellular level, their functional connections, and analyzed the clonal lineage of persister and resistant cells using a RGB multicolor barcoding system.

## Materials and Methods

### Reagents

Temozolomide was purchased from Interchim (Montluçon, France), and all other drugs were purchased from Sigma (Saint Louis, MO) unless stated otherwise. All cell culture products were obtained from Life Technologies (Carlsbad, CA).

### Cell Culture

U251 cells were cultured in DMEM (4.5 g/l glucose) enriched with 10% FCS with 100 U/ml penicillin, 100 μg/ml streptomycin, and 2 mM l-glutamine. Cells were maintained in 5% CO_2_ at 37°C. The U251 cell line was certified by Eurofins Genomics (Ebersberg, Germany). U251 cells used were *mycoplasma*-free as described in Gratas et al. (2014).

### Cytotoxicity Assay and Cell Counts

MTT assays were performed as previously described in Rabé et al. (2020). Viable cell counts were performed using the Countess optics and image automated cell counter (Invitrogen, CA), after trypan blue staining. Data are presented as the percentage of viable cells after treatment compared to untreated cells.

### RGB Cells

With the human U251 cell line, LeGO-RGB lentiviral vectors were used as color-guided clonal cell trackers. LeGO-C2 (Addgene: 27,339), LeGO-V2 (Addgene: 27,340), and LeGO-Cer2 (Addgene: 27,388) plasmids were employed to produce lentiviral vectors coding for red, green, and blue fluorescent proteins, respectively, according to the method previously described in Gambera et al. (2018). The RGB color mix was achieved using a MOI of 0.75, which corresponds to an equimolar transduction efficiency of 50% per lentiviral vector 3 days after transduction. Cell transduction efficacy and colored cell distribution and stability were followed by FACS.

### FACS Analysis

The cells were trypsinized, counted, and then resuspended in 1 ml PBS containing 1% BSA. Data acquisition was performed with the BD FACSymphony (BD Biosciences^®^) and BD FACSDiva 8.5 analysis software. The Cerulean protein was excited at 405 nm and detected with a 525/50 filter. The Venus protein was excited at 488 nm and detected with a 530/30 filter, and the Cherry protein was excited at 561 nm and detected with a 610/20 filter. The cells were initially sorted according to their size (forward scatter, FSC) and their complexity (side scatter, SSC). The U251-RGB cell population was selected, and doublets were excluded. Using three different strategies, namely, a green strategy allows for the visualization in a dot plot containing the blue, red, and magenta cells; a red strategy allows for the visualization in a dot plot containing the blue, green, and cyan cells; and a blue strategy allows for the visualization of red, green, and yellow cells. The amount of unlabeled (R^−^G^-^B^-^), red (R^+^G^−^B^-^), blue (R^−^G^-^B^+^), green (R^−^G^+^B^−^), magenta (R^+^G^−^B^+^), yellow (R^+^G^+^B^−^), cyan (R^−^G^+^B^+^), and white (R^+^G^+^B^+^) cells were then quantified. The average number of events of each color was calculated and then transcribed as a percentage.

### Gene Knockdown Using siRNA

ON-TARGETplus—SMARTpool Human siRNA (CHI3L1: # L-012568-01, KLK5: #L-005916-00, #HB-EGF: L-019624, and FAT2: # L-011270-00, Dharmacon, CO) were transfected at a final concentration of 15 nM in U251 cells with Lipofectamine RNAi MAX (Life Technologies) according to the recommended protocol. siRNA scramble (scr) (sc-37007, Santa-Cruz, TX) was used as a negative control. Cells were reverse transfected on day 1. A second classical transfection was performed on day 4.

### Gene Expression Assay

RNA reverse transcription was performed using Maxima First Strand cDNA Synthesis Kit for RT-qPCR (Thermo Scientific, MA). Quantitative real-time PCR (qPCR) assays were performed in triplicate using the Perfecta™ SYBR^®^ Green FastMix™, Low ROX™ (Quanta BioSciences, CA), and the real-time thermal cycler qTower (Analytik Jena AG, Germany). Quantitative fold change was calculated as previously described (Rabé et al., 2020) using RPLPO, TATA, and HGPRT as housekeeping genes.

### Transcriptomic and Single-Cell Analysis

Transcriptomic analyses are based on previously published results (Rabé et al., 2020). Gene expression analysis in single cells was measured using the C1 Single-Cell Auto Prep System coupled with the real-time PCR reader BioMark HD (Fluidigm, CA, USA) according to Fluidigm recommendations in untreated cells and in cells treated with 50 µM temozolomide for 4, 9, 12, and 16 days. Single-Cell Preamp IFC of 10–17 μM and 17–25 µM was used, respectively, for U251-S (untreated), U251-R (resistant), and U251-TR (persister population: day 4, day 9, and day 12). The results were analyzed using the Singular Analysis Toolset Software provided by Fluidigm.

### Data Availability statement

RNAseq data are available on NCBI trace Archive. PRJNA479416 www.ncbi.nlm.nih.gov/bioproject/?term=prjna479416.

## Results

### Resistance to Temozolomide Is Not Associated With a Specific Pre-Existent Subpopulation in U251 Cells

**FIGURE 1:**
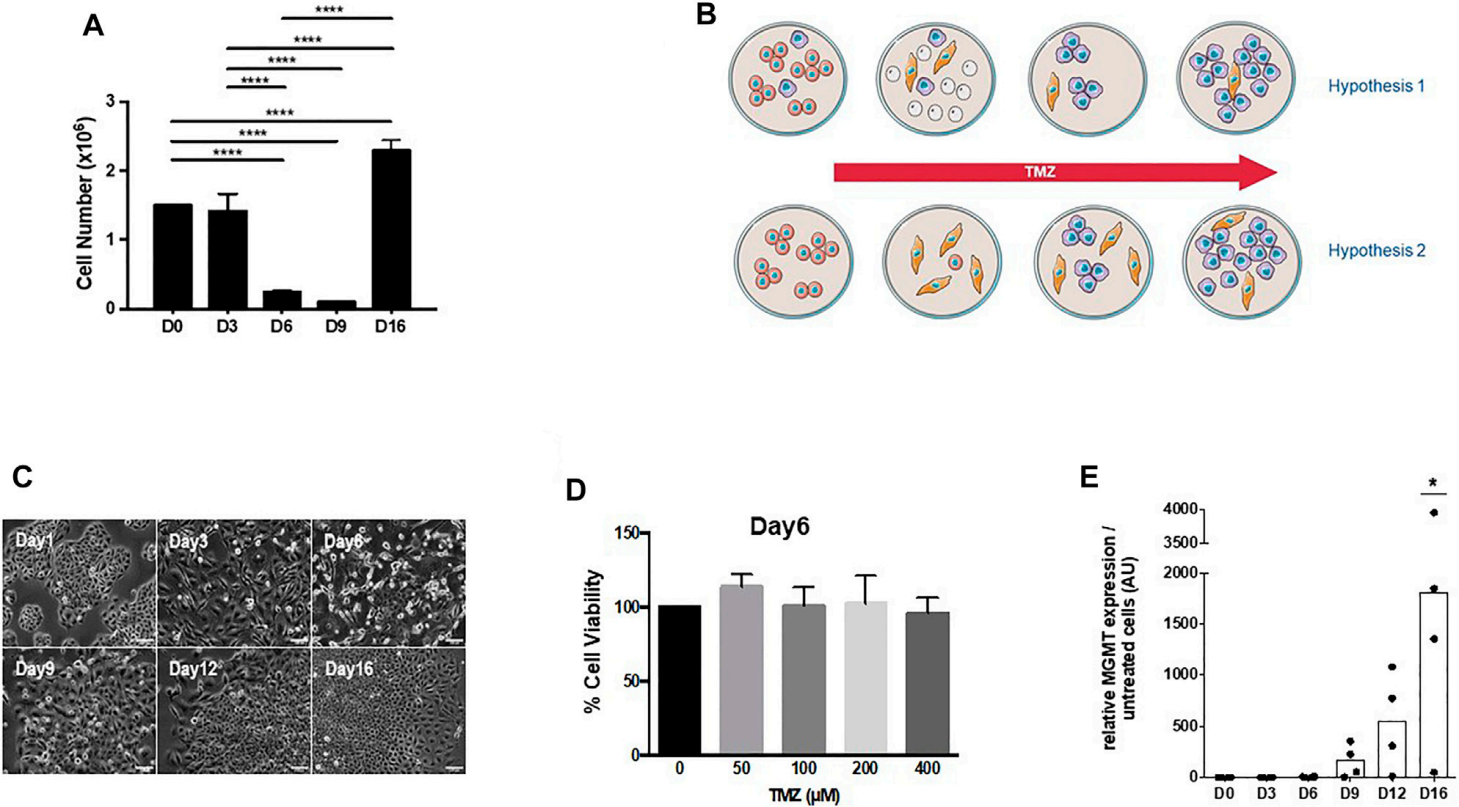
**(A)** U251 cells were treated every 3 days for 3 weeks with 50 μM TMZ, a dose relevant to the clinical situation. A maximum cell death rate was observed after 3 days of TMZ exposure (data not shown), and cell numbers were measured by MTT assay and continued to decrease until day 9 before resuming proliferation as observed previously (Rabé et al., 2020). **(B)** Two models for the acquisition of temozolomide (TMZ) resistance in U251 cells. Hypothesis 1: a small proportion of U251 cells expresses MGMT either in a stable or intermittent way or restricted to slow-cycling cells (thus escaping MGMT quantification by Q-PCR). This subpopulation is resistant to TMZ and progressively acquires fast-growing properties and overcomes the original population. Hypothesis 2: cells in the original population do not express MGMT, but some cells are intrinsically or transiently resistant to stress induced by the drug. Slow-cycling cells from this population acquire the expression of MGMT and a fast-growing pattern to become the predominant population. **(C)** Morphologically, surviving U251 cells after 3-day TMZ treatment exhibited flattened cell bodies with long cellular extensions (day 3 to day 9) and in a second phase cells, started to grow as colonies (day 9 to day12) before a third phase (i.e., after day12) when cells recolonized the entire dish. **(D)** Tolerant/persister cells are not sensitive to TMZ even at very high doses: U251 cells, treated for 6 days with 50 μM TMZ, were treated with different concentrations of TMZ with no effect on survival. **(E)** Kinetics of MGMT expression in U251 cells exposed to TMZ. MGMT expression was determined by qPCR as published earlier (Rabé et al., 2020).

We have previously established a simple model for studying temozolomide resistance in GBM using the human glioma cell line U251, which lacks MGMT expression (Rabé et al., 2020). Treatment with temozolomide did not produce an immediate effect on U251 at 50 µM (a clinically relevant concentration), and a peak in cell death was observed after day 3 with a minimal survival of around 10% of the initial population (Figure 1A). Resistance to temozolomide appears to be associated with the expression of MGMT after a latency period, which is consistent with glioma response *in vivo* as reported in the literature (Kitange et al., 2009). A cell cycle arrest (G2/M blockade) was observed between day 3 and day 9 after treatment; before the cells resume after day 12, rapid cell cycling and concomitant expression of MGMT were observed as previously reported (Rabé et al., 2020). Based on the response lag before the appearance of the fast-growing temozolomide-resistant, MGMT-expressing U251 cells, we have established a mathematical model for the acquisition of resistance using the expression of MGMT as a marker (Rabé et al., 2020). Two classical hypotheses were explored: i) the clonal selection and ii) the adaptive/selection model. In the first scenario, a small subpopulation of cells with high levels of resistance due to MGMT expression is present in the untreated population; and in the second case, the induction of a genetic drift amplified by the presence of the drug that would lead to the selection of resistant cells (Figure 1B). Our previous results and mathematic model (Rabé et al., 2020) favor the existence of a transient state from sensitive to resistant cells similar to persister cells. We found that the expression of MGMT was not detectable during the early stages of treatment but increased gradually between day 9 and day 12 (Rabé et al., 2020). Nonetheless, cells treated with temozolomide were not sensitive to the drug from day 4 to day 9 (Figure 1C), even at high doses (Figure 1D), and in the absence of MGMT expression (Figure 1E) but showed low clonogenicity (Supplementary Figure S1). However, it cannot be excluded that a small population of slow-cycling cells with the capacity of expressing MGMT could be present in the initial U251 cell population, which would be thus both activated and selected upon temozolomide treatment. Our mathematic model predicted that these cells would represent less than 0.02% of the total cell population (Rabé et al., 2020), a similar range to that estimated for persisters in cancer (Sharma et al., 2010). A second question would be how homogeneous are these pre-resistant and persister populations (i.e., mono- or polyclonal populations).

**FIGURE 2:**
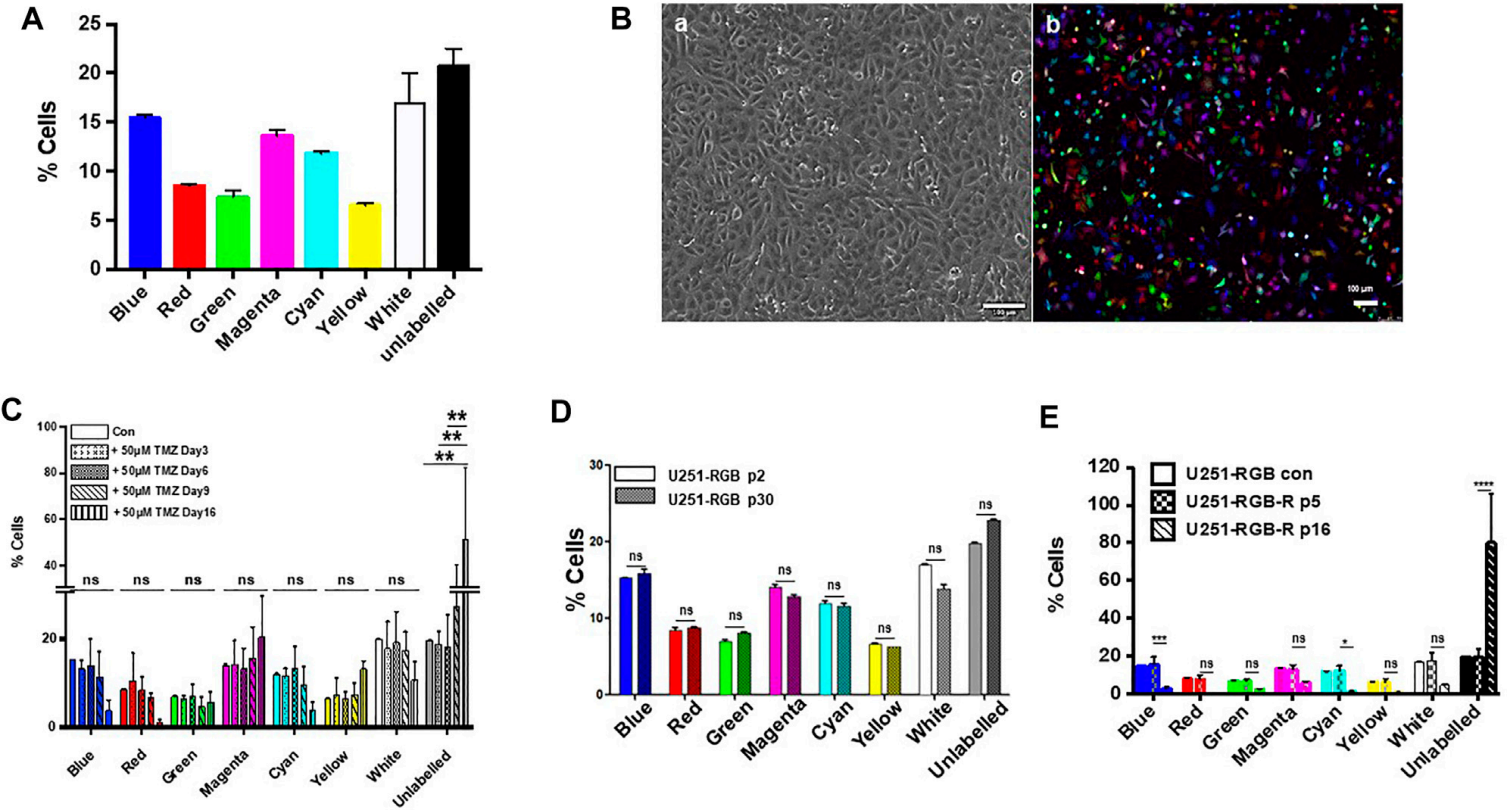
**(A)** FACS analysis of U251-RGB cells at passage two showing the distribution of the different colored populations. **(B)** Images of U251-RGB cells under phase contrast **(A)** and under confocal microscopy **(B)**. **(C)** FACS analyses of untreated U251-RGB cells or cells treated for 3, 6, 9, and 16 days with 50 µM TMZ. No significant statistical differences were found between the different samples (days of treatment) except for day 16 which shows an increase in unlabeled cells. **(D)** FACS analyses of U251-RGB cells at passage 2 (p2) and passage 30 (p30); the latter passage corresponds to 5 months in culture without TMZ. **(E)** FACS analyses of U251-RGB cells vs. U251-RGB resistant cells at passage 5 (p5) and passage 16 (p16). This suggests that acquisition of resistance is accompanied by a loss in labeled cells. All statistical analyses were obtained from experiments performed at least three times.

To address these questions, we used a multicolor lentiviral genetic barcode to generate RGB-labeled U251 cells. The U251-RGB cells were obtained after the simultaneous transduction of the cells with three lentiviral gene ontology (LeGO) vectors encoding red, green, or blue fluorescent proteins (Supplementary Figure S2). Since our conditions favor single-cell multiple transductions, the RGB-marked cells display at least eight different color conditions consistent with a normal distribution (Figure 2A). Using confocal microscopy, the fluorescence of U251-RGB appears to be randomly distributed, but cells exhibiting low, medium, and high fluorescent generate an expression spectrum (Figure 2B). The multicolor lentiviral genetic barcode labeling has been widely used to track single cell progeny during culture expansion or clonal dominance during tumor progression (Gambera et al., 2018).

Since, the putative MGMT-expressing population in the initial U251 population is expected to be extremely small as MGMT was not detected by PCR until day 9, we postulated that it was unlikely that these cells could be distributed in all eight different colors. We treated the U251-RGB cells with 50 μM temozolomide, and the cells behaved similarly to the unlabeled U251 cells with a cell death phase observed after 3 days of treatment, followed by a latency phase, and then a rapid extension period (Supplementary Figure S1). The distribution of fluorescence was followed for 16 days after temozolomide treatment, and no significant change in the color distribution was observed upon the treatment, as illustrated in Figure 2C. The only noticeable difference was an increase in unlabeled cells observed in cells at day 16, which express MGMT and thus are resistant to temozolomide. We observed that after several passages, the color distribution, which was stable in sensitive cells, was altered in resistant U251-RGB cells even in absence of temozolomide (Figures 2D,E). This is in agreement with an increase in genetic instability and clonal diversity in resistant cells as observed previously (Rabé et al., 2020). Taken together, our result suggests that it is unlikely that a minor subpopulation existed in the untreated population. Another possibility was that these cells could be refractory to lentiviral transduction, and thus the increase in unlabeled cells could be the clonal expansion of pre-existing resistant cells. However, this assertion would not account for the percentage of the different sub-populations of RGB-labeled cells observed in cells treated for 16 days by temozolomide (Figure 2C). A similar reasoning could apply to the persister cell population since no change in color distribution was observed between day 4 and day 9 after drug treatment. We conclude from these experiments that no pre-existing resistant or persister subpopulation was present in our untreated U251-RGB cells.

### Single-Cell Analysis and Interdependence of the Four-Gene Persister Signature

As previously reported (Rabé et al., 2020), a longitudinal RNA-sequencing study allowed us to determine a persister-like signature in U251 cells responding to temozolomide treatment. The four genes implicated in this signature were: i) *CHI3L1*, which has an unclear biological function but is associated with inflammation and extracellular tissue remodeling (Zhao et al., 2020); ii) *FAT2* gene encodes for an atypical cadherin, which is involved in tissue remodeling through cell–cell interactions, and most likely functions as a cell adhesion molecule, controlling cell proliferation (Fulford and McNeill, 2020); iii) *HB-EGF* encodes for a soluble and membrane-bound ligand of the EGF receptor, which has multiple roles in both auto- and paracrine-EGFR signaling; and iv) *KLK5* encodes for a peptidase, which has been shown to activate several extracellular peptidases, thereby controlling the release and activity of growth factors and other extracellular matrix proteins (Nauroy and Nyström, 2019). Quite strikingly, all these genes encode for proteins with extracellular functions and have implications in wound healing and tissue remodeling. Of note, this signature was also partially found in other glioma cell lines in response to temozolomide, and TCGA glioblastoma database analysis for *KLK5*, *FAT2*, *CHI3L1*, and *HB-EGF* expression showed that alteration of these genes impacts Kaplan–Meier estimate of overall survival and disease/progression-free (Rabé et al., 2020).

**FIGURE 3:**
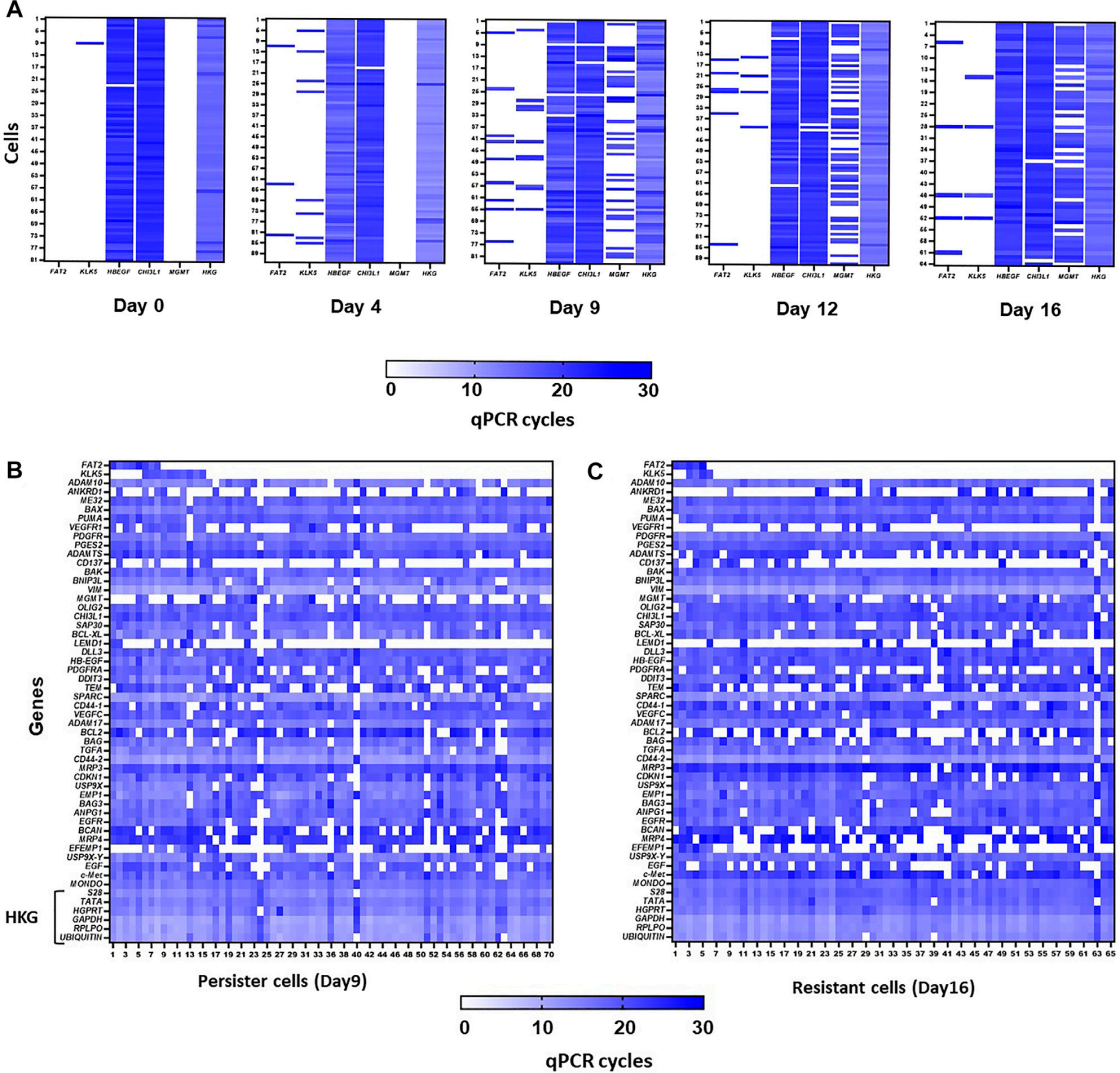
**(A)**. Heatmaps from single-cell analysis using C1 Fluidigm technology. Representation of qPCR cycles for *FAT2*, *KLK5*, *CHI3L1*, *HB-EGF*, and *MGMT* genes, at days 0, 4, 9, 12, and 16 post-treatments of U251 cells with 50 µM TMZ. Ct average of six housekeeping genes is represented in column HKG. **(B,C)** Heatmap from single-cell analysis using C1 Fluidigm technology. Representation of qPCR cycles in persister **(B)** and resistant **(C)** cells for a panel of genes involved in different pathways. HKG: panel of housekeeping genes.

Single-cell analysis by the Fluidigm C1/HD BioMark quantitative PCR technique (Rabé et al., 2020) showed that two of the markers (*HB-EGF* and *CHI3L1*) were present in all cells and that their expression was increased during the persister state (Figure 3A). On the other hand, *FAT2* and *KLK5* were only expressed after day 4 of temozolomide treatment and more importantly, in different cells until day 12 and day 16 (Figure 3A). However, when MGMT is expressed in almost all cells, the expression of the four genes was found within the same cells (Figure 3A). These results suggest that the expression of the markers does not occur in a subpopulation of surviving cells after 4 days’ treatment, at least before the acquisition of MGMT. Next, we analyzed 84 genes representative of different cellular functions including apoptosis, and stemness along with *KLK5, FAT2, CHI3L1*, and *HB-EGF* on day 9 and day 16 after temozolomide treatment. The gene signature in *FAT2*- or *KLK5*- expressing vs. non-expressing genes in cells at day 6 (i.e., assimilated to the persister stage) did not reveal a distinct signature (Figure 3B). Similar analysis at day 16 after temozolomide treatment (i.e., resistant U251 cells) indicated that cells expressing *FAT2* or *KLK5* are not significantly different from non-expressing ones (Figure 3C).

**FIGURE 4:**
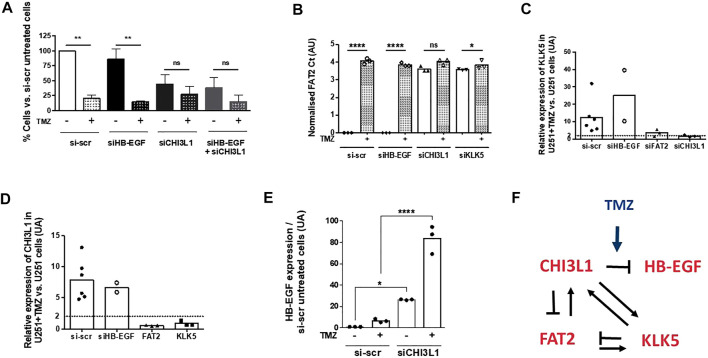
**(A)** Effect of siRNA silencing of *KLK5*, *FAT2*, *CHI3L1*, and *HB-EGF* on cell survival in the absence or in the presence of 50 µM TMZ (7 days treatment). Q-PCR quantification of *FAT2* **(B)**, *KLK5* **(C)**, and *CHI3L1* **(D)** in U251 cells treated with siRNA directed against each of the four genes in our persister signature with or without TMZ treatment. **(E)** Expression of *HB-EGF* is increased only by *CHI3L1* silencing and is enhanced by TMZ treatment. **(F)** Representation of the regulation loops between the four genes of our persister signature.

To further examine the interconnection between the four genes, we inhibited the expression of each gene in U251 cells using siRNA technology in order to examine the consequence on the expression of the other genes. As previously described (Rabé et al., 2020), the silencing of *CHI3L1*, *KLK5*, *FAT2*, and *HB-EGF* affected basal U251 cell survival but not the response to temozolomide (Figure 4A). The silencing of HB-EGF had no consequence on the expression of *FAT2*, which was affected by the silencing of *CHI3L1* and *KLK5*. Temozolomide had no effect on the expression of *FAT2* under these conditions (Figure 4B). The expression of *KLK5* was affected by the silencing of both *FAT2* and *CHI3L1* and not by that of *HB-EGF* (Figure 4C). *CHI3L1* expression was reduced by a knockdown of *KLK5* and *FAT2* but not by that of HB-EGF (Figure 4D). The expression of *HB-EGF* was unaffected by the silencing of *KLK5* and *FAT2* in the presence or in the absence of treatment (data not shown). On the other hand, a knockdown of *CHI3L1* increased the expression of *HB-EGF* transcripts, and this was amplified by temozolomide (Figure 4E). Altogether, these results suggested that the expression of the four genes was essential for cell survival even in the absence of temozolomide and that their expressions were interconnected (Figure 4F).

## Discussion

The question of treatment resistance is central in cancer as too many patients die from non-responding recurrent tumors. It is, therefore, of the utmost importance to stop the development of resistant populations in cancers, especially when the treatment itself leads to more aggressive phenotypes through the induction of genome instability, mutations, somatic copy-number alterations, and epigenetic changes (Wu et al., 2021). A tolerant/persister state that precedes and/or accompanies the development of resistance has been evidenced in many cancers treated with different drugs (Swayden et al., 2020; De Conti et al., 2021). The slow-cycling persister cells appear to share common features but may be different depending on the different treatments and from one type of cancer to another (Swayden et al., 2020; De Conti et al., 2021). Nonetheless, these cells appear to be attractive new targets in cancer providing precise mechanisms that could be targeted. In a recent study, we have shown using a GBM cell line that common features with other drug-tolerant/persister cells could be found in glioma (i.e., metabolism, epigenetics, and apoptosis reprogramming) but that only targeting epigenetics was efficient to eliminate persisters (Rabé et al., 2020). Here, we show using a multicolor lentiviral genetic barcode that persister cells and resistant cells are likely to be induced by the treatment rather than present in the pre-existing naïve cell line. The four-gene signature identifies a set of genes encoding for proteins with extracellular activities, which are not expressed in the same cells, at least until day 12 (Figure 3). From the silencing studies (Figure 4), an interaction network can be suggested, which could, through the remodeling of the microenvironment, facilitate the survival and the emergence of resistant cells. Indeed, the silencing of *CHI3L1*, *KLK5*, and *FAT2* affects the survival of U251 cells even in absence of temozolomide suggesting that these genes play a fundamental role in the survival program. The mechanisms implicated are still to be discovered and to be extended to other cell lines and/or to primary cultures. Nonetheless, our results suggest that paracrine factors might be a key element for the survival of GBM cells after temozolomide treatment, its acquisition of drug tolerance/persister traits, and the selection of temozolomide-resistant clones.

## Data Availability

The datasets presented in this study can be found in online repositories. The names of the repository/repositories and accession number(s) can be found at: https://www.ncbi.nlm.nih.gov/, PRJNA479416.
