# Supplementary material for: Cellular Heterogeneity and Cooperativity in Glioma Persister Cells Under Temozolomide Treatment
Source: Front Cell Dev Biol. 2022 May 25;10:835273. doi: 10.3389/fcell.2022.835273 (PMC9174429; doi:10.3389/fcell.2022.835273)
Supplement: Supplementary file 1 [file DataSheet1.docx]

**Supplementary figures: Rabé et al**


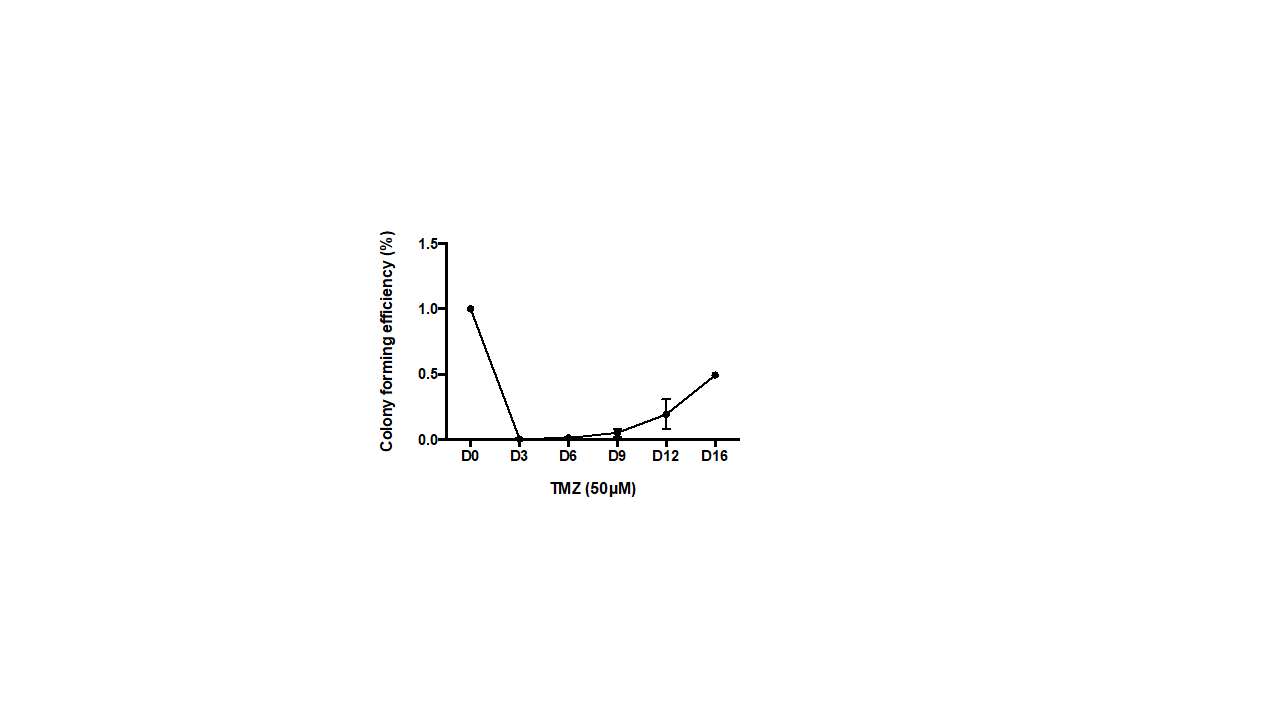


**Supplementary Figure 1:** U251 cells, pretreated with 50 µM TMZ at indicated times, were seeded at appropriate densities in 6 well dishes. At least two dilutions of cells were used for each TMZ treatment time. Cultures were incubated for 1 to 2 weeks for colony formation. Then cells were stained with 0,5% crystal violet and colonies were counted using ImageJ (NIH). Colony forming efficiency of TMZ-treated cells was calculated as: [(number of clones/number of plated cells)/(number of wells)*Plated Efficiency for untreated U251 cells].


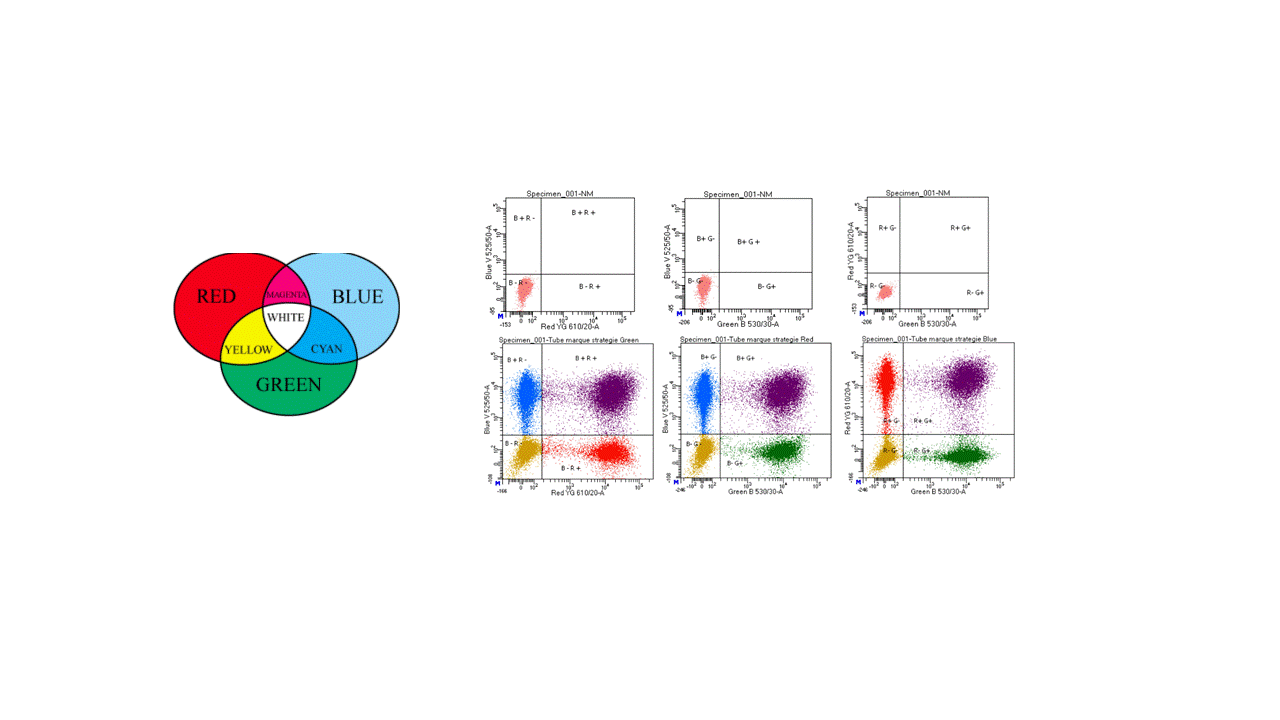


**B.**

**A.**

**Supplementary Figure 2**: **A.** Spectrum of the different colors constituting U251-RGB cells. **B.** Image of the distribution of the different cell populations of U251-RGB cells after FACS analyses.
